# A review of the development of interventional devices for mitral valve repair with the implantation of artificial chords

**DOI:** 10.3389/fbioe.2023.1173413

**Published:** 2023-06-02

**Authors:** Tingchao Zhang, Yichen Dou, Rifang Luo, Li Yang, Weiwei Zhang, Kangmu Ma, Yunbing Wang, Xingdong Zhang

**Affiliations:** ^1^ National Engineering Research Center for Biomaterials, College of Biomedical Engineering, Sichuan University, Chengdu, China; ^2^ Hangzhou Valgen Medtech Co., Ltd., Hangzhou, China

**Keywords:** mitral regurgitation, artificial chordal implantation, expanded polytetrafluoroethylene (ePTFE), chordal ruptures, transapical, transcatheter, interventional devices

## Abstract

Mitral regurgitation (MR) was the most common heart valve disease. Surgical repair with artificial chordal replacement had become one of the standard treatments for mitral regurgitation. Expanded polytetrafluoroethylene (ePTFE) was currently the most commonly used artificial chordae material due to its unique physicochemical and biocompatible properties. Interventional artificial chordal implantation techniques had emerged as an alternative treatment option for physicians and patients in treating mitral regurgitation. Using either a transapical or a transcatheter approach with interventional devices, a chordal replacement could be performed transcatheter in the beating heart without cardiopulmonary bypass, and the acute effect on the resolution of mitral regurgitation could be monitored in real-time by transesophageal echo imaging during the procedure. Despite the *in vitro* durability of the expanded polytetrafluoroethylene material, artificial chordal rupture occasionally occurred. In this article, we reviewed the development and therapeutic results of interventional devices for chordal implantation and discuss the possible clinical factors responsible for the rupture of the artificial chordal material.

## 1 Introduction

**FIGURE 1:**
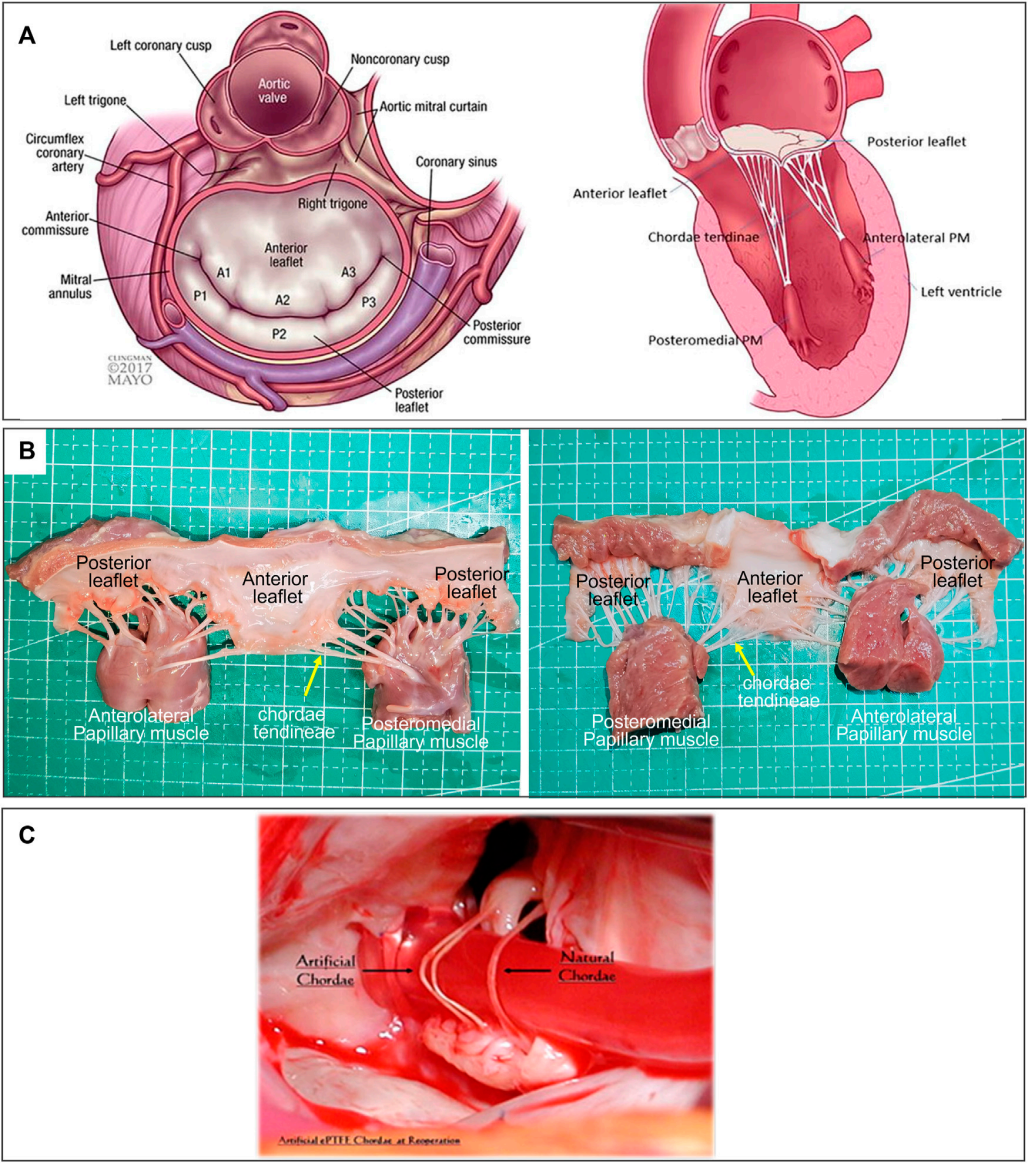
**(A)** Atrial and sagittal views of mitral valve. Left, P1-A1 was anterolateral, P3-A3 was posteromedial. Right, Sagittal view showing subvalvular supporting structures. A indicates anterior; P, posterior; and PM, papillary muscle (Ali et al., 2020). **(B)** Mitral Valve Anatomical Map. **(C)** Comparison of artificial and native chordae, reoperation after 12 years (Salvador et al., 2008).

Mitral regurgitation (MR) was a common heart valve disease in clinical practice and was characterized by the backflow of blood from the left ventricle to the left atrium during diastole (Enriquez-Sarano et al., 2009; Xu et al., 2022). A large number of patients require urgent treatment (Li et al., 2016). The mitral apparatus consisted of the mitral annulus, leaflets, commissures, chordae tendineae, posterior left atrium, LV free wall, and papillary muscles; dysfunction of any of these components may lead to mitral valve pathology, including MR (Figure 1A, Figure 1B) (Ali et al., 2020). Among them, degenerative MR (DMR) caused by structural lesions of the valve itself, such as mitral valve stenosis or prolapse due to redundancy or chordal rupture, was one of the main etiologies of MR (Soulat-Dufour and Addetia, 2020). Different treatment modalities were chosen clinically depending on the etiology of MR, which was divided into mitral valve replacement and repair. With better long-term outcomes, fewer valve-related complications, and lower mortality (Gillinov et al., 2008), mitral valve repair has emerged as a treatment option for patients with DMR.

Mitral valve repair mainly consists of annuloplasty (96.1%), leaflet resection (58.9%), and prosthetic chordae repair (29.2%) (Gammie et al., 2018b). With the development of technology, “preservation without resection” was the principle of mitral valve repair surgery. This allows complete preservation of lobular tissue and subvalvular structure to better preserve left ventricular function. A meta-analysis of 6,046 patients in 17 studies of mitral valve repair by Ibrahim et al. (2012) showed that the “preserved method” was superior to the “resected method.”

Artificial chordae surgery repairs mitral prolapse or flail leaflets by replacing the diseased chords with a string-like material. This approach does not affect the surrounding valve structure and was more in keeping with the physiological anatomy of the valve. It has become the standard of care for DMR (Bortolotti et al., 2012b).

Although surgical mitral valve repair has a good long-term therapeutic effect, it must be performed under the conditions of thoracotomy and extracorporeal circulation, which were more invasive. Compared with surgical thoracotomy, minimally invasive interventional repair devices can be performed under non-extracorporeal circulation and continous heartbeat, with the advantages of less trauma and faster postoperative recovery (Ahmed et al., 2021).

The artificial chord intervention instrument was a minimally invasive interventional technique based on surgical mitral valve chordal repair, which may be divided into transapical chordal repair and transcatheter chordal repair according to the surgical approach. It could capture the prolapsed or tethered leaflet under ultrasound and/or x-ray guidance and implant the prosthetic chordae. It could also be readjusted to the appropriate length to restore leaflet alignment and reduce or eliminate MR (Fiocco et al., 2019). Many artificial chordae interventional devices have been developed based on this principle (Maisano et al., 2013), and some of them have entered the clinical or commercial application stage.

This review reports the development of artificial chordae materials, the principles of two routes of artificial chordae intervention devices, implantation methods, force differentials, and their clinical application effects and failure modes.

## 2 Development history of artificial chordae materials

The selection of materials was particularly critical for chordal replacement. The selection and research of artificial chordal materials began as early as the mid-20th century (David, 2004). To have a stable function under the complex physiological environment and long-term stress conditions *in vivo*, the ideal material should have the following properties: 1) High tensile strength and inelasticity to avoid fracture or deformation under the action of stress. 2) Resistance to calcification and mineral deposition to prevent hardening or fracture of the artificial chord after calcification. 3) Good fatigue properties, resistant to creep or fracture. 4) Fine, pliable, and capable of retaining this property long after implantation. 5) Porous structure that allows tissue growth to form a stable fibrous envelope and endothelial layer. 6) Good biocompatibility, resulting in producing an acceptable inflammatory response and resistance to thrombosis (Kunzelman and Cochran, 1990; David, 2004; Casado et al., 2012).

Initially, silk sutures, polyester sutures, polypropylene sutures, and pericardium were used to be artificial chords; however, they all had certain limitations and were abandoned due to changes in length and thrombosis after implant (January et al., 1962; Kay and Egerton, 1963; Morris et al., 1964; David, 2004). Among the pericardial materials, thickening, and stiffening, and contracture were found early after implantation of the autologous pericardium (Rittenhouse et al., 1978); while for allogeneic pericardial materials, a certain degree of flexibility was maintained in the early stage of implantation, but they showed insufficient long-term effects with fibrous hyperplasia, calcification, thickening and stiffness 3 years later (Frater et al., 1983). These materials must experience more than hundreds of millions of cardiac contractions. Because they had less flexibility, tensile strength, histocompatibility, and durability, they were difficult to fully meet the application requirements, so they have not been used and commercialized in large-scale clinical applications.

It was reported that expanded polytetrafluoroethylene (ePTFE) sutures were the gold standard in current clinical practice for the use of artificial chordae (David, 2022). The ePTFE suture was a microporous, non-absorbable monofilament suture made of PTFE that can stretch and expand, and also had many other unique properties. The molecular formula of ePTFE was the same as PTFE. Its chemical structure was very stable and resistant to hydrolysis and aging. The expanded and stretched ePTFE suture had good flexibility and tensile strength. It also had good biocompatibility and carries negative surface charges consistent with endothelial cells, making it less prone to thrombosis (Wang G. et al., 2022). The surface of the microporous structure was conducive to tissue growth, and the degree of inflammation was lower than that of the multifilament braided sutures (Nistal et al., 1990).

Gore’s GORE-TEX^®^ ePTFE product was commercially used in clinical practice and was approved by the FDA in 1985. Two major sizes were shown in Table 1.

**TABLE 1:** GORE-TEX® ePTFE suture.

Specification	Diameter (mm)	Tensile strength (kg)
CV-4	0.404	1.79
CV-5	0.321	1.62

When ePTFE was used for artificial tendons, it was found that after a few months of implantation, the surface was covered with fibers and endothelial tissue. This discovery was a major stimulus for ePTFE research. The two pioneers who extensively tested ePTFE on animals were Frater and Revuelta (Pomar et al., 2013). Revuelta et al. (1989) used ePTFE sutures as artificial chordae to replace the chordae of the ovine mitral valve. They resected one or two marginal chordae of the anterior leaflet of the mitral valve in 35 sheep and replaced them with a double-armed, pledget-supported, expanded polytetrafluoroethylene suture. The 30 surviving animals were hemodynamically studied and euthanized 3, 6, 9, 18, and 24 months after surgery. None of the sheep experienced mitral insufficiency. All specimens had normal mitral valves without thrombosis. The polytetrafluoroethylene suture remained pliable and was incorporated into the anterior leaflet and papillary muscle. Scanning and transmission electron microscopy showed that the suture was completely covered by a sheath of tissue with a collagen structure remarkably similar to that of native chordae. There was no evidence of calcification in the new chordae. This reproducible and safe technique may greatly simplify the difficult repair of chordae anomalies. This result was also confirmed in an authoritative article on chordal replacement published by Zussa (1996). Based on discussions with Frater, David began using the ePTFE suture as an artificial chord in the clinical treatment of DMR patients in 1989, and has done so for over 30 years (David, 1989). David et al. (2013) reviewed the long-term outcomes of 606 patients with ePTFE artificial chordal between 1986 and 2004. During the 18-year follow-up period, the cardiogenic mortality rate related to valvular disease was 8.5%. The reoperation rates at 1 year, 10 years, and 18 years were 1.4%, 5.3%, and 9.8%, respectively, and recurrent moderate/severe MR was 1.5%, 12.2%, and 32.5%. Hata et al. (2014) also reported on the long-term outcomes of 224 patients who received ePTFE artificial chordae between 1988 and 2013. Over 20 years, the mortality rate related to cardiac and valvular disease was 3.1%. The reoperation rates at 1, 10, and 20 years were 7%, 16%, and 26%, respectively, and recurrent moderate/severe MR were 9%, 18%, and 41%. These studies demonstrated that the clinical efficacy of early and long-term use of ePTFE suture as an artificial chord to repair DMR valve prolapse was effective.

The long-term durability of ePTFE artificial chordae was closely related to their biocompatibility. This has been confirmed by the histopathology of many ePTFE chordae taken removed during reoperations. The ePTFE sutures implanted *in vivo* remained macroscopically smooth and pliable, with a morphology close to that of native chordae (Figure 1C) (Salvador et al., 2008). Histopathologic examination showed that protein infiltrated into the internal microporous structure of ePTFE, and the surface was covered with fibrous tissue and endothelium by histopathological examination. These tissue-material composite artificial chordae were formed by the interaction between the material and the organism. It improved the suture and reduced the inflammatory response and thrombosis, ensuring its long-term stability in the body (Hata et al., 2014).

## 3 Implantation methods and differences between artificial chords

**FIGURE 2:**
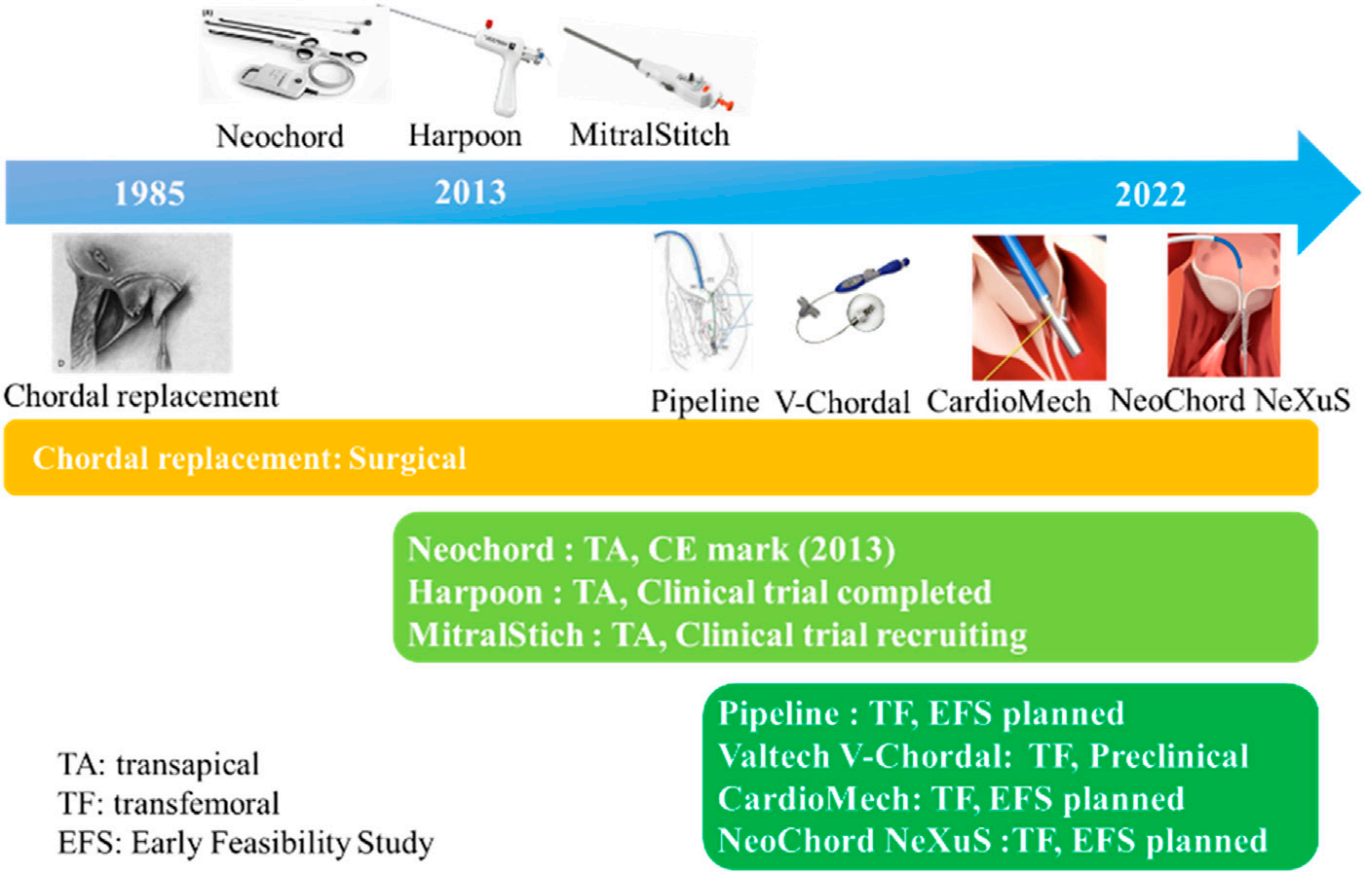
The development process of artificial chorda implantation technology.

ePTFE sutures have been used as artificial chordae for more than 35 years with proven durability and clinical efficacy. Currently, ePTFE sutures were used in both surgical and minimally invasive interventional devices. There were three methods of implantation: 1) surgical thoracotomy; 2) minimally invasive transapical implantation; and 3) transfemoral vein/atrial septal implantation (David, 2004; Fiocco et al., 2019). The advantages and disadvantages of the above repair techniques were listed in Table 2 and Figure 2 summarized the development process of artificial chordae implantation technology and various representative devices.

**TABLE 2:** Advantages and disadvantages of the interventional repair techniques.

	Surgical	Transapical	Transcatheter
Material	ePTFE	ePTFE	ePTFE + metal anchor
Therapy method	Conventional thoracotomy and cardiopulmonary bypass	Minimally invasive, beating heart	Interventional and transseptal treatment
Risk and prognosis	The surgical risk was relatively high, and the postoperative prognosis was relatively slow	Risk was lower than surgical, and the prognosis was faster than surgical	Risk was relatively low, and the prognosis was relatively fast
Applicable patients	Younger patients with low surgical risk	Patients unable to undergo conventional surgical procedures	Patients unable to undergo conventional surgical procedures
Location of prolapse	Posterior leaflet prolapses, anterior leaflet prolapses or bileaflet prolapse	Isolated posterior leaflet prolapses	Isolated posterior leaflet prolapses

### 3.1 Surgical thoracotomy implantation

**FIGURE 3:**
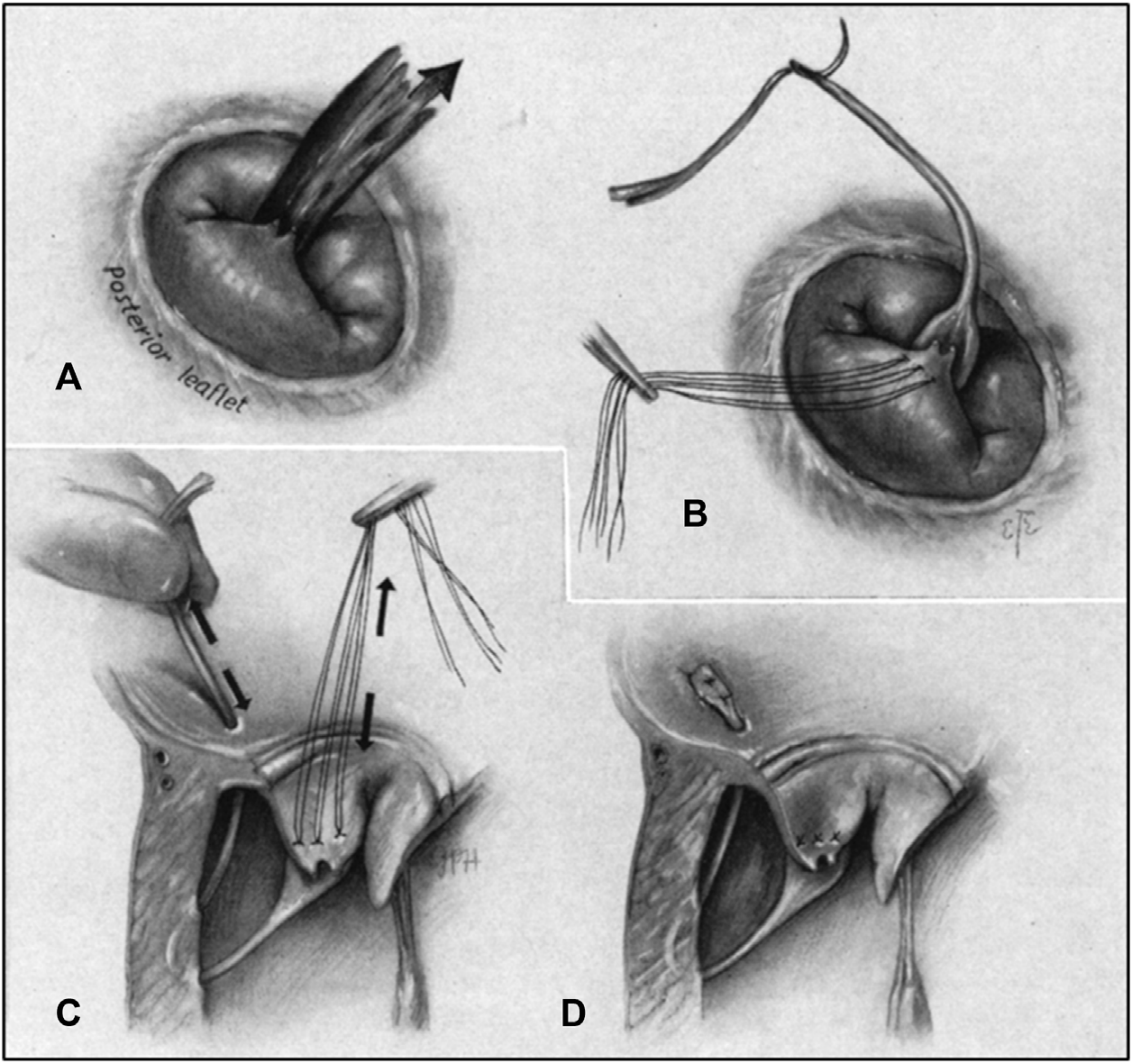
**(A)** Regurgitant jet produced by chordal rupture attached to posterior leaflet as seen from right thoracotomy. **(B)** Prosthetic chorda attached to flailing leaflet. **(C)** Pulley stitch completed in the ventricular wall. Tension adjusted on chorda, restoring leaflet position. **(D)** Prosthetic chorda anchored at the atrial wall and excess length excised (Morris et al., 1964).

The surgical method of artificial chordae implantation was to stop the heart beating with the support of extracorporeal circulation, then fully exposed the heart through a median sternotomy. The mitral valve was exposed through the right atrium-atrial septal incision or a combined right atrium-atrial septal-left atrial apex incision, and artificial chordae were implanted under direct visualization (Figure 3) (Morris et al., 1964).

Due to cardiac arrest, the suture length cannot be adjusted in real time to determine whether to eliminate regurgitation, and the smoothness of the ePTFE suture made tying the knot difficult, Therefore, surgeon experience was very important (Madhurapantula et al., 2020). The position of the chordae anchor was at the edge of the valve leaflet and the papillary muscle, which corresponded to the physiological and anatomical structure and had a good long-term effect. However, it was very traumatic for patients, and the postoperative recovery period could be as long as 6 weeks (Oh et al., 2020). A study of 449 patients who underwent mitral valve surgery from January 1995 to December 1999 showed that the operative mortality rate was 4.4%. Therefore, it may not applied to many patiens at high surgical risk patients due to poor cardiac function, high comorbidity, advanced age, and other factors (Bech-Hanssen et al., 2003).

### 3.2 Minimally invasive transapical implantation

**FIGURE 4:**
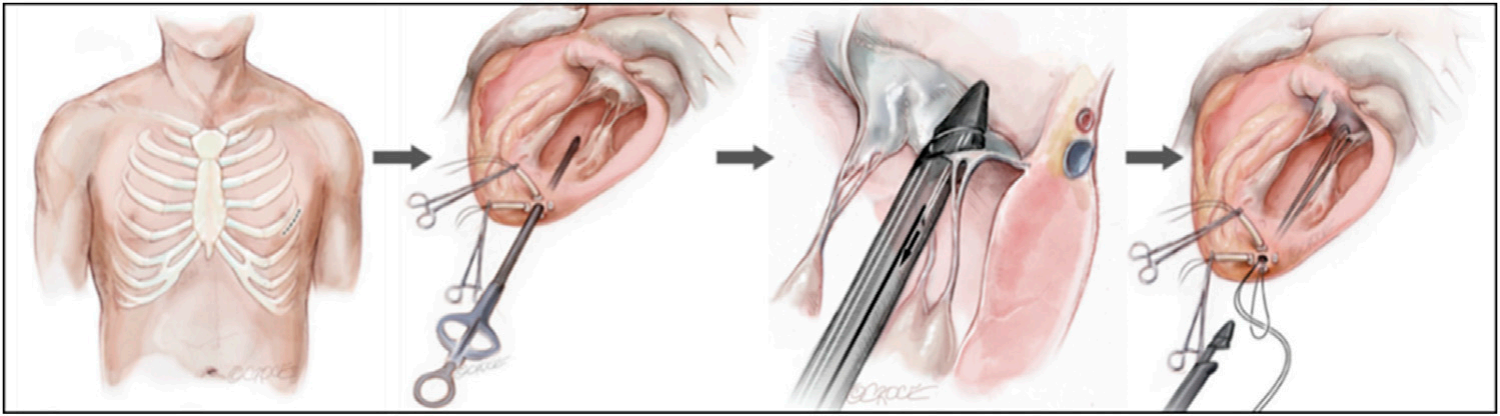
Transapical NeoChord mitral valve repair (Colli et al., 2018a).

Minimally invasive apical implantation was the implantation of ePTFE artificial chordae under 2D and 3D transesophageal echocardiographic (TEE) guidance without cardiopulmonary bypass while the heart continued to beat. In this method, only a small incision of approximately 4 cm was made in the fifth or sixth intercostal space to expose the cardiac apex of the heart, followed by deployment of the apical load and a ventriculotomy to establish the apical access (Figure 4) (Colli et al., 2018a). The artificial chordae were implanted and anchored to the edge/root of the leaflets and the apical part of the epicardium, guided by TEE. As the heart beats continuosly during the procedure, the suture length can be adjusted in real-time to determine the optimal length. Because the access of the transapical minimally invasive repair device was short, it had advantages such as short operation time, easy operation and high success rate. Although this approach was less traumatic for patients than surgical thoracotomy, it was prone to complications such as pericardial adhesions or left ventricular rupture due to damage to the apical and pericardial tissues (Blumenstein et al., 2012; Simões Costa et al., 2021).

### 3.3 Transcatheter implantation

**FIGURE 5:**
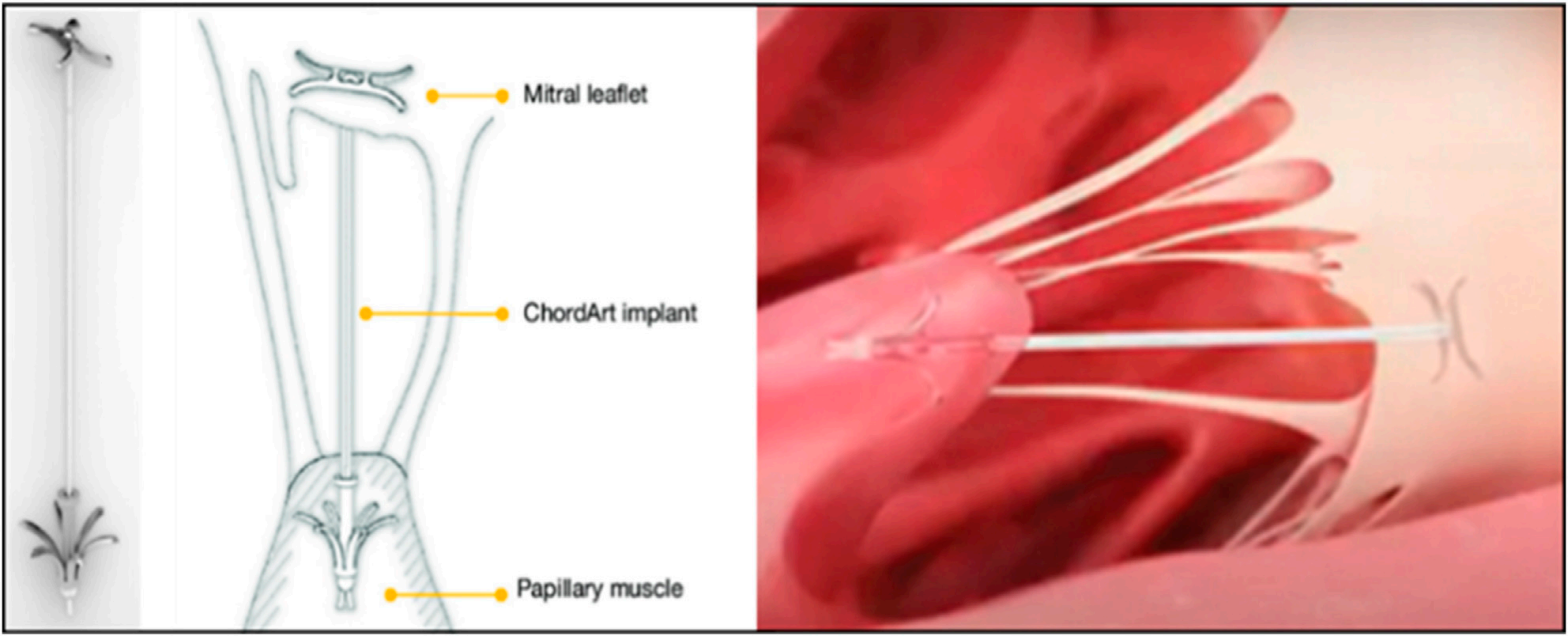
ChordArt implant. There were nitinol leaflets and papillary muscle anchors with an ePTFE chord (Rogers and Bolling, 2021; Weber et al., 2021).

Transcatheter implantation meant that the device entered through the femoral vein, punctures the interatrial septum, and then entered the left atrium and ventricle to complete the implantation of artificial chordae anchored at the edge of the valve leaflets and the papillary muscle under the guidance of echocardiography and digital subtraction techniques (Figure 5) (Rogers and Bolling, 2021; Weber et al., 2021). This approach allowed real-time adjustment of chordal length without the need for cardiac arrest or extracorporeal circulatory support. It also required no thoracic or cardiac incision, which was minimally traumatic for the patient, who recovered quickly and was discharged 2–3 days postoperatively (Oh et al., 2020).

However, the success rate of transcatheter chordae implantation was low due to the length of the approach, the complexity of the mitral valve anatomy, the difficulty of the technique, and the narrow indication. It was currently in the early stages of development.

### 3.4 Differences of surgical, transapical and transcatheter repair

#### 3.4.1 How many and where to place the artificial chordae

Depending on the extent and location of the prolapsed area, the number and placement of the artificial chordae may vary. One study found that the artificial chordae tendineae technology could preserve the effective opening area of the valve leaflet to the maximum extent, and could effectively cope with large-scale pathological changes by inserting a plurality of artificial chordae tendineae, better preserving the systolic and diastolic function of the left ventricle (Perier et al., 2008). A few studies have reported outcomes after mitral valve repair with artificial chordae including surgical repair and transapical repair (Table 3).

**TABLE 3:** Differences of mitral valve repair therapeutic strategy.

Therapeutic strategy	Repair method	Patient number	Area of lesion	Number of artificial chordae implanted	References
Surgical	Posterior leaflet repair using chordae alone	192	PML	1–6	Lange et al. (2010)
	Double-armed approach using ePTFE chordae and Carpentier resection	608	PML, AML, Bi-ML	4–8	Salvador et al. (2008)
	Loop technique	129	PML	3-4	Falk et al. (2008)
	Simple chordae replacement	74	PML, AML, Bi-ML	2–12	Kobayashi et al. (2000)
Transapical Neochord	Transapical off-pump MVr	10	PML (58.3%), AML (26.7%), Bi-ML (25%)	3–6	Kavakli et al. (2016)
	Transapical off-pump MVr	213	PML (90.6%), AML (5.2%), Bi-ML (4.2%)	3–4	Colli et al. (2018b)
	Transapical off-pump MVr	7	PML	3–4	Grinberg et al. (2019b)
	Transapical off-pump MVr	7	PML	3–5	Heuts et al. (2018)
	Transapical off-pump MVr	6	PML	3 or 3+	Kiefer et al. (2018)
	Transapical off-pump MVr	4	PML	2–3	Wang et al. (2019)
Transapical Harpoon	Transapical off-pump MVr	11	PML	3–5	Gammie et al. (2016)
	Transapical off-pump MVr	65	PML	0–7	Gammie et al. (2021)

AML, anterior mitral leaflet; PML, posterior mitra leaflet; Bi-ML, bilateral mitral leaflet (Figures 1A, B).

#### 3.4.2 The position of the artificial chordae tendineae fixation

Surgical implantation of artificial chordae was a technique that was gaining acceptance. This technique involved suturing one or more artificial chordae to the head of a papillary muscle and the other end of the artificial chordae to the prolapsed leaflet (Aubert and Flecher, 2010). The length of the artificial chordae was appropriately adjusted to maintain the free edge of the anterior mitral leaflet at the same level as the free edge of the posterior leaflet below the annular plane to obtain an adequate area of apposition (Zussa et al., 1997). All chords attached directly to the papillary muscle (PM). The posteromedial PM gave chords to the medial half of both leaflets (i.e., posteromedial commissure, P3, A3 and half of P2 and A2) (Figure 1A). Similarly, the anterolateral PM chords attached to the lateral half of the MV leaflets (i.e., anterolateral commissure, A1, P1 and half of P2 and A2). During surgical mitral valve repair, the physician arrached another segment of the artificial chordae tendineae to the papillary muscle following the normal anatomy.

In minimally invasive apical implantation, ePTFE artificial chordae were implanted on the leaflet and the chordae were stretched until adequate leaflet coaptation was achieved and then all chordae free ends were secured to the LV wall (Colli et al., 2018a). Annabel M et al. performed an *in vivo* biomechanical study of apical versus papillary neochordal anchoring for mitral regurgitation. Force tracings were compiled for each class of chordae at baseline, after prolapse, and after both repair techniques. Baseline forces were recorded for primary (0.18 N ± 0.08 N) and secondary (0.94 N ± 0.31 N). Echocardiographic and hemodynamic data confirmed that the repairs restored physiological hemodynamics. Forces on the chordae and neochord were lower with papillary fixation than with apical fixation (*p* = 0.003). In addition, the maximum rate of change of force on the chordae and neochordae was higher for apical fixation than for papillary fixation (mean difference of 4.7 N/s, *p* = 0.028). Annabel M highlighted the impact of the anchoring position of artificial neochordae and might help guide strategies to increase durability. Although the development of minimally invasive and percutaneous devices for mitral valve repair was benefiting patients by providing superior survival and freedom from reoperation, adjustments to reduce leaflet stress were essential to fully utilize these new devices.

#### 3.4.3 Chordal replacement in combination with partial leaflet resection

Chordal replacement with ePTFE strings to correct leaflet prolapse in patients with DMR was initially used for prolapse of the anterior leaflet, but over the years it was been used to correct prolapse of all segments of the MV. The use of Gore-Tex sutures as artificial chords to replace native chordal has largely been used to correct prolapse of the anterior leaflet, commissural areas, and posterior leaflet with heights of 20 mm or less. However, large, voluminous posterior leaflets with prolapse must first be treated with partial resection followed by chordal replacement. Patients with advanced myxomatous degeneration often had large posterior leaflets and associated posterior displacement of the posterior mitral annulus. In these cases, the posterior leaflet was usually trimmed to a height of 15–18 mm, and the posterior leaflet was sutured back to the endocardium of the left ventricle with running 4-0 polypropylene sutures (David et al., 2020). In David’s study, isolated chordal replacement was used to correct prolapse in 186 (24.9%) patients and combined with leaflet resection in 560 (75.1%) patients. In their practice, chordal replacement with ePTFE sutures did not provide better results than MV repair using the established techniques of leaflet resection and chordal transfer, but it has dramatically increased the likelihood of MV repair in patients with MR due to leaflet prolapse.

Finite element results (Choi et al., 2017) showed that both repair techniques revealed reduced leaflet prolapse, decreased stress concentration, and restored leaflet coaptation. While neochordoplasty demonstrated further improved leaflet coaptation and superior posterior leaflet mobility, leaflet resection showed more uniform leaflet stress distributions. Virtual MV repair simulation has the ability to predict and quantify biomechanical and functional improvement after MV repair.

It could be said that chordal replacement with ePTFE sutures to correct mitral valve leaflet prolapse, either alone or in combination with leaflet resection all allowed a low probability of mitral valve reoperation and recurrent mitral regurgitation.

#### 3.4.4 Concomitant combination therapy (COMBO)

A cohort of 595 (278 women, mean age 65 ± 16 years) consecutive patients with isolated mitral valve prolapse, with comprehensive clinical, rhythmic, Doppler echocardiographic, and consistent mitral annular disjunction assessment. The presence of mitral annular disjunction was common [*n* = 186 (31%)] in patients with mitral valve prolapse, generally in younger patients, and was not random but was independently associated with severe myxomatous disease with bileaflet mitral valve prolapse and marked leaflet redundancy (Essayagh et al., 2021). Javier G reported that 744 consecutive patients with degenerative mitral regurgitation and prolapse underwent mitral valve surgery from January 2002 to December 2010 (Castillo et al., 2012). All patients underwent mitral valve repair with concomitant annuloplasty with a median ring size of 32 mm, and 175 patients (23.5%) underwent PTFE chordoplasty. Mitral ring annuloplasty could reverse annular dilation and restored the zone of coaptation in appropriately selected patients (Yap et al., 2021). If neochordae were implanted, concomitant annular reduction might help restore better systolic leaflet coaptation and reduced neochord forces. For transapical and transcatheter devices, there were several types of transcatheter mitral valve repair (TMVr) that tareted the leaflets, annulus and chordae. Concomitant combination (COMBO) therapy of TMVrs was rarely used as a treatment and there were very few publications about this therapeutic strategy (Yokoyama et al., 2023), only transcatheter edge-to-edge repair has been combined with other interventional repair methods to treat mitral regurgitation. One could speculate that the COMBO approach might be a better option for patients suffering from severe MR with a selected anatomy where the combined devices could best play out their advantages, simultaneous mitral ring annuloplasty and PTFE chordoplasty as in surgical treatments.

## 4 Artificial chordae interventional implantation devices

Artificial chordae interventional repair was based on the proven chordae implantation technique, which used special devices to repair the valve while the heart was beating. The following section described the principle, implantation methods, force differences, and therapeutic effects of the artificial chordae interventional devices.

### 4.1 Transapical artificial chordal intervention implantation devices

At present, the apical chordae repair devices that have entered the clinical stage included NeoChord DS1000 (NeoChord, United States), Harpoon TSD-5 (Edwards Life Science, United States), MitralStitch (Hangzhou Valgen Medtech Co., Ltd., China), and other products. The technical difference was mainly in the method of anchoring the chordae. NeoChord and Harpoon have received CE certification (Savic et al., 2018).

#### 4.1.1 Neochord DS1000 system

**FIGURE 6:**
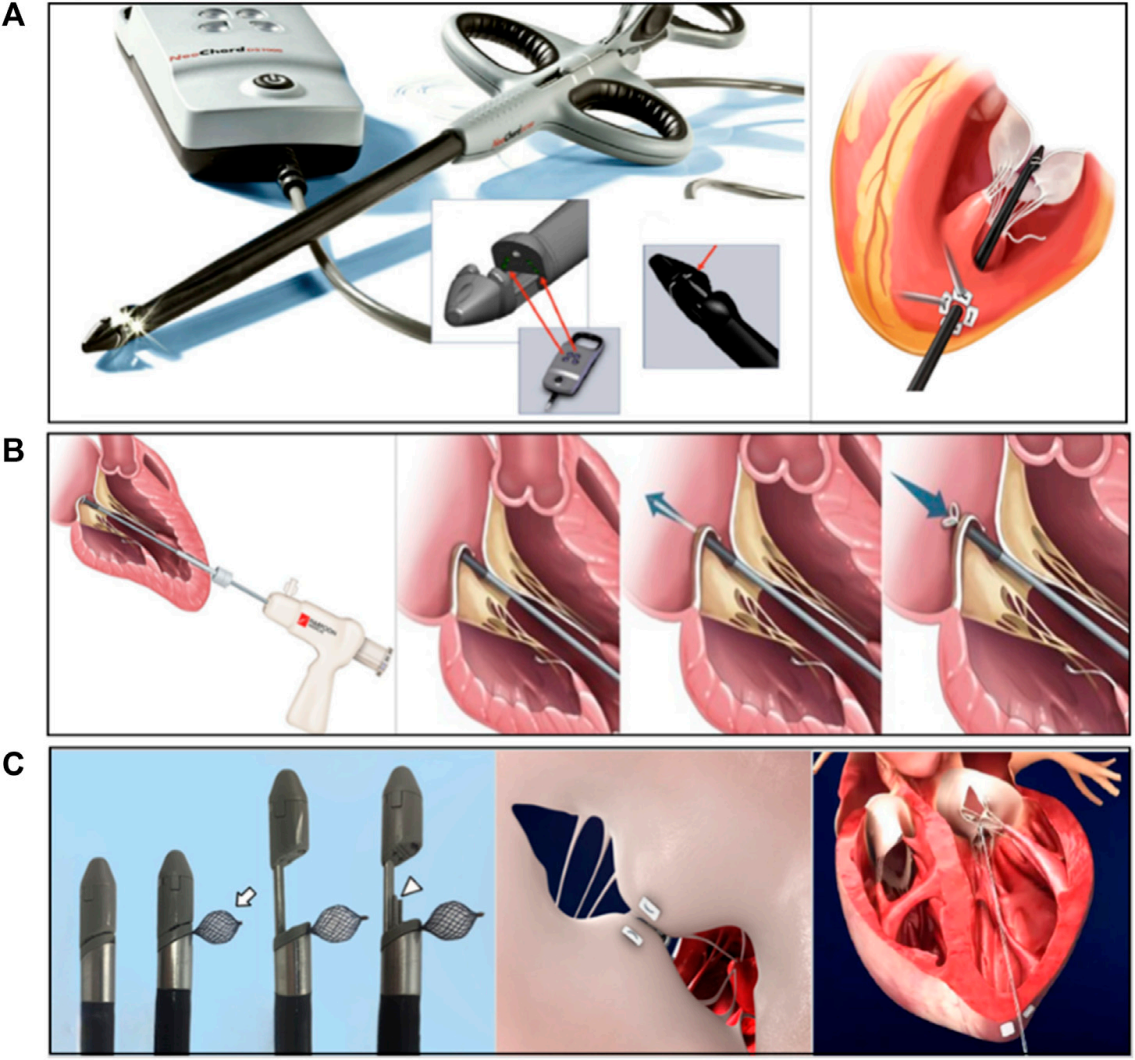
**(A)** The NeoChord DS 1000 system (Rucinskas et al., 2014; Fernando et al., 2020), **(B)** The Harpoon TSD-5 system (Gammie et al., 2016), **(C)** The MitralStitch system (Wang et al., 2018). Courtesy of (http://www.dinovamedtech.com).

The Neochord DS1000 system was the first CE-marked transapical minimally invasive chordae repair product with over 1,700 patients implantated and was currently in FDA pivotal clinical trials. The system consisted of the Gore-Tex CV-4 size ePTFE suture. The conveyor has the following functions: valve leaf capture, optical fiber sensor detection of valve leaf capture, and artificial chordal anchor valve leaf (Figure 6A) (Rucinskas et al., 2014; Fernando et al., 2020). The procedure consisted of entering the left ventricle through the apical channel, crossing the mitral valve into the left atrium under TEE guidance, and then opening the collet to capture the leaflet. After the fiber optic sensor confirmed the capture, the leaflet was punctured and one end of the artificial chord was anchored to the leaflet edge, and the other end was withdrawn from the heart and fixed apically after real-time adjustment of the chord length to eliminate regurgitation under TEE guidance (Rucinskas et al., 2014).

Gerosa et al. (2021) reported a 3-year follow-up experience of a patients who underwent NeoChord DS1000 surgery for severe DMR due to single/bipartite leaflet mitral valve prolapse or flail between November 2013 and June 2019. The study enrolled 203 patients with severe DMR, of whom 200 (99%) had successful surgery (implantation of ≥2 pairs of chords and MR ≤ 1); patient survival rates and primary endpoints (successful surgery, MR ≤ 2+ and no major adverse events) were 91.2%, 89.7%, and 81.2% at 1, 2, and 3 years, respectively. Survival and re-intervention rates at 3 years were 94.0% and 6.4%, respectively. This suggested that the NeoChord DS1000 system for minimally invasive repair early to mid-term DMR was safe, effective, and reproducible. Early clinical and echocardiographic results showed reduction in MR and significant improvement in symptoms with good left ventricular remodeling and low reintervention rates. However, the long-term effects needed to be confirmed with more clinical data and longer follow-up. The authors also examined the effect of different anatomic types (type A, isolated central posterior leaflet prolapse and/or flail; type B, posterior multisegmental prolapse; type C, anterior and/or bi-leaflet prolapse; and type D, paracommissural prolapse and/or flail and/or significant leaflet and/or annular calcifications) on repair outcomes, with probabilities of achieving effective end points of 88%, 83%, 72%, and 57%, respectively. This suggested that transapical chordal repair might be more appropriate for patients with posterior leaflet prolapse.

D'Onofrio et al. (2022) reviewed the treatment outcomes comparing the NeoChord DS1000 device group to the surgical group. A propensity analysis selected 88 matched pairs. Kaplan–Meier analysis showed similar 5-year survival in the 2 groups. The ratio of moderate/severe MR in patients who underwent instrumentation versus surgery was: MR > 2+ (42.4% vs. 15.4%) and MR ≥ 3+ (21.6% vs. 10.3%), respectively; the reoperation rate was 21.1% vs. 8%, with a significant difference. However, in type A patients, moderate/severe MR (63.9% vs. 74.6% and 79.3% vs. 79%) and reoperation rates (79.7% vs. 85%) were close with no significant difference. Demonstrating that transapical beating-heart mitral valve chordae implantation can be considered as an alternative treatment for degenerative mitral regurgitation, especially in patients with isolated P2 (central posterior leaflet) region prolapse.

#### 4.1.2 Harpoon system

The Harpoon system was another transapical chordal repair device (Figure 6B) (Gammie et al., 2016) that differed from the Neochord DS1000 in that the chord was anchored to the root of the valve leaflet. The Harpoon system (Edwards Lifesciences, Irvine, CA, United States) was a 14-Fr, echo-guided, transapical chordal replacement device that allowed for the implantation of multiple and adjustable PTFE chordae. The Harpoon device was inserted and precisely navigated to the target. The prolapsed segment was punctured and an ePTFE chord was knotted on the atrial side of the prolapsed segment. The cord was externalized through the introducer (Gackowski et al., 2022). Safety and feasibility were demonstrated in the initial feasibility study in 30 patients with severe degenerative MR. At 1 month, MR was mild or less in 89% of patients and moderate in 11% of patients. At 6 months, MR was mild or less in 85% of patients, moderate in 8% of patients, and severe in 8% of patients (Gammie et al., 2021). Gammie et al. (2021) reported the results of a 1-year CE Mark clinical trial of the Harpoon system, mean study follow-up 1.4 ± 0.6 years, 1-year clinical follow-up was 100%, 1-year echo core lab follow-up for 52 patients at 1 year was 100%. 65 patients were enrolled and 62 (95%) achieved procedural success. Only two patients required conversion to surgery and one patient discontinued the procedure. Total procedure time was 126 ± 36 (72–222) min, and the introducer time was 42 ± 18 (18–126) min. Intraoperative blood loss was 272 ± 182 (50–949) ml and this data was collected for TRACER CE Mark study only (*n* = 51). The number of chords implanted was 4.0 ± 1.1 (0–7). During the perioperative period (from procedure to discharge), the rate of mortality, stroke rate, renal failure, and atrial fibrillation rate were 0%, 0%, 0%, and 18%, respectively. Atrial fibrillation was pooled only for those without baseline atrial fibrillation (*n* = 50). At 1 year, 2 of the 62 patients died (3%) and 8 (13%) others required reoperation. At 1 year, 98% of patients with Harpoon cords were in New York Heart Association class I or II, and mitral regurgitation was none/trace in 52% (*n* = 27), mild in 23% (*n* = 12), moderate in 23% (*n* = 12), and severe in 2% (*n* = 1). Favorable cardiac remodeling outcomed at 1 year included decreased end-diastolic left ventricular volume (153 ± 41 to 119 ± 28 ml) and diameter (53 ± 5 to 47 ± 6 mm), and a mean transmitral gradient of 1.4 ± 0.7 mmHg. This early clinical experience with the Harpoon beating heart mitral valve repair system demonstrated encouraging early safety and performance.

#### 4.1.3 MitralStitch system

**FIGURE 7:**
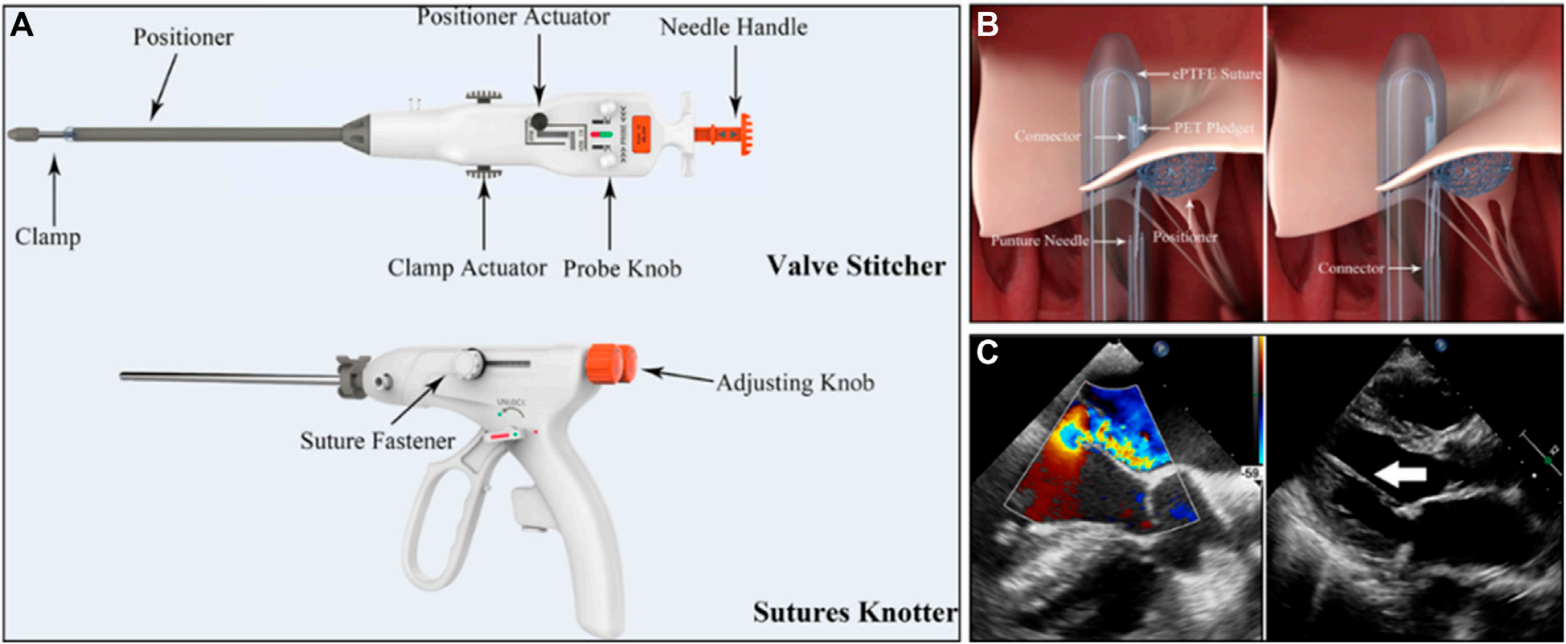
**(A)** The MitralStitch™ system device, Valve Stitcher and Sutures Knotter. The suture and pledget were preloaded in the head of the Valve Stitcher. **(B)** Mechanism of artificial chordae implantation by Valve Stitcher. **(C)** (Left) Intraprocedural two dimensional and color TEE image showing severe MR due to P2 region leaflet prolapse. (right) TTE view showing the implanted artificial chordae (arrow) (Wang S. et al., 2022).

MitralStitch was the first product to offer both edge-to-edge repair and chordae repair. For edge-to-edge repair, a pair of chordae was implanted in each anterior and posterior leaflet, and the two chordae were locked together with titanium staples, resulting in a double-hole structure in the anterior and posterior leaflets (Figure 6C) (Wang et al., 2018). It was similar to Neochord for chordae repair (Figure 7A). The chord was implanted at the edge of the leaflet and anchored to the apex of the heart (Figure 7B). Wang S. et al. (2022) reported the first-in-human (FIH) experience with MitralStitch, which was successful in all 10 patients with severe MR, including 9 patients who underwent chordae repair, and 1 patient who received chordae repair and edge-to-edge repair. At discharge, MR decreased from severe to trace in 5 patients and was mild in the other 5 patients (Figure 7C).

In conclusion, the transcatheter apical chordae repair device was easy to operate and had a high technical success rate. Theoretically, this technique can treat not only high-risk patients who were ineligible for surgery but also young patients with low surgical risk, Which could largely replace traditional surgery and has a promising application prospect. Appropriate patient inclusion criteria were also a key factor in achieving the best results. In some complex MR anatomies, surgical repair was superior. In type A patients with isolated P2 prolapse/flail, minimally invasive implantation via the apical approach was close to the early and mid-term results of surgery. Therefore, it was an ideal entry point for transapical chordal implantation as an adjunct to surgery. However, the transcatheter approach remained the ultimate approach for artificial chordae, as the apical approach was still traumatic and carried the risk of long-term pericardial adhesion.

### 4.2 Transcatheter intervention artificial chordae repair devices

**FIGURE 8:**
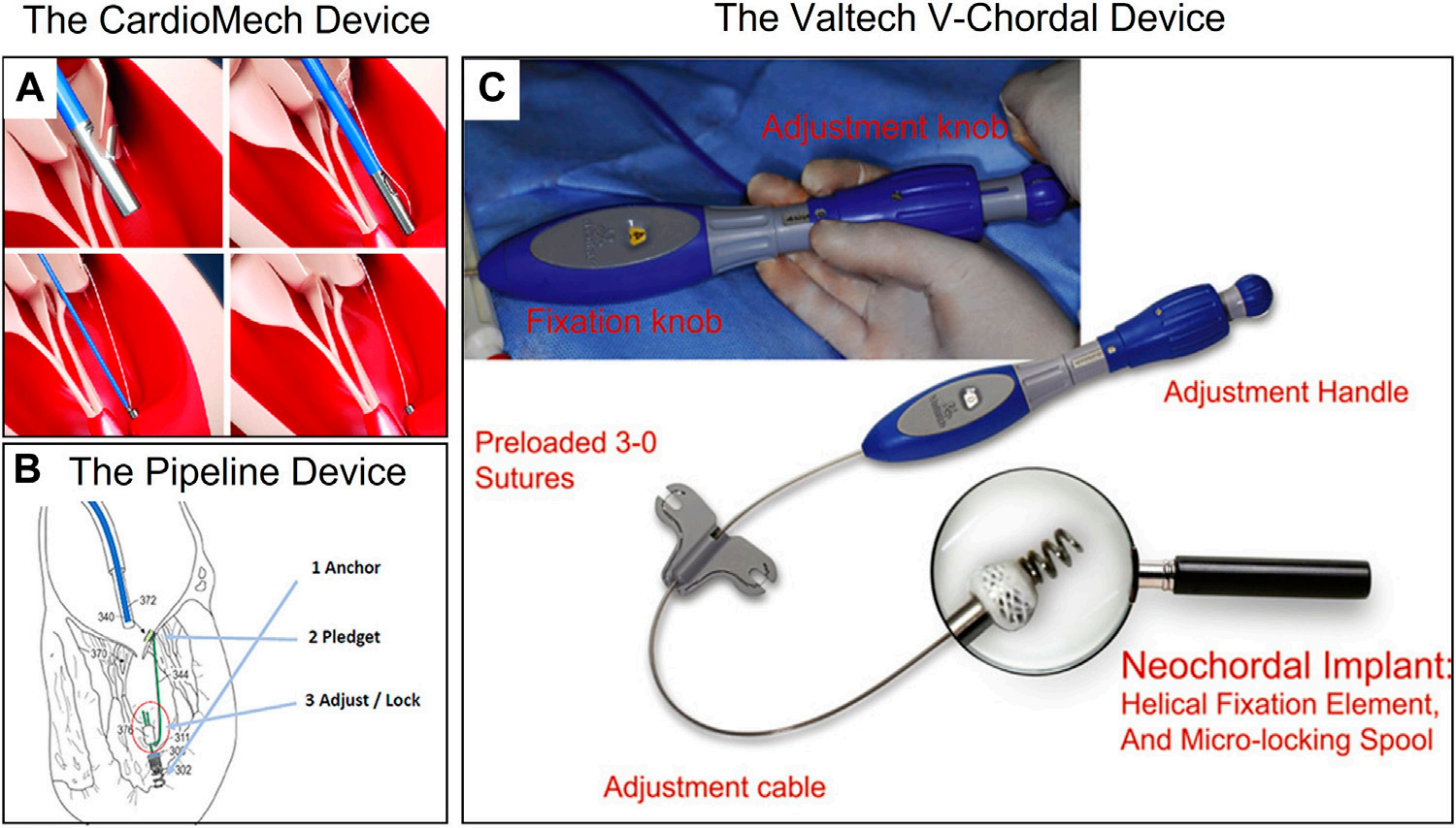
**(A)** The CardioMech Device. Courtesy of (CardioMech, Oslo, Norway) (Rogers and Bolling, 2021). **(B)** The Pipeline Device. Courtesy of (Pipeline Medical Technologies, Inc. a wholly-owned subsidiary of W.L. Gore & Associates, Inc., Santa Rosa, California) (Fiocco et al., 2019). **(C)** The Valtech V-Chordal Device. Courtesy of (Valtech, Or Yehuda, Israel) (Maisano et al., 2011).

Transcatheter chordae repair devices had attracted much attention due to their lower trauma and better compliance with the physiological and anatomical structure. However, due to their technical difficulties, the products were currently in animal experiments or early clinical stages, including NeoChord NeXuS (NeoChord, United States), CardioMech (CardioMech, Norway) (Figure 8A) (Rogers and Bolling, 2021), and Pipeline (Gore, USA) (Figure 8B) (Fiocco et al., 2019), which have performed animal experiments and early clinical experiments. The transcatheter chordae repair device was composed of implants and a delivery system. The implants included ePTFE sutures (artificial chordae), papillary muscle anchor pieces, and leaflet anchor pieces connected at both ends. The delivery system included the large sheath for spacer puncture, the adjustable curved middle sheath, and the chordae implantation components (leaflet suture device, papillary muscle anchor device, chordae length adjustment, locking and cut-off devices, etc.). In addition, stents were included to assist in device stabilization procedures.

Unlike transapical artificial chordae devices, transcatheter devices required the implantation of metal anchors to hold sutures to the papillary muscle, which may carry the risk of damaging the papillary muscle. Animal studies of the Neochord NeXuS system reported at the Transcatheter Cardiovascular Therapeutics (TCT) conference showed that the anchor nail on the papillary muscle was covered by fibrous tissue and endothelium at 90 days. Overall, the papillary muscle healed well overall and no obvious necrosis was observed. Maisano et al. (2011) compared the healing difference between metal screw anchors and surgical sutures anchoring the papillary muscle in the V-chordal system (Figure 8C) through *in vivo* experiments in sheep, and both showed good healing of at 90 days. Animal experiments with the Pipeline system also confirmed that the metal anchors did not damage the papillary muscle. These experiments provided preliminary confirmation of the *in vivo* safety of metal anchors for anchoring the papillary muscle.

Transcatheter chordae repair devices were still in the early stages of development due to their technical difficulty, and only a few clinical trials were reported. Rogers et al. (2020) reported in 2020 the first human trial of the Pipeline system (Figure 8B) in a 56-year-old male patient with severe MR due to prolapse in the P2 region, in which two artificial chords were successfully implanted transcatheterly and attached to a papillary muscle base anchor, eliminating MR intraoperatively. However, the papillary muscle anchor was found to be dislodged prior to discharge, resulting in recurrent MR, and the patient ultimately underwent mitral valve replacement. The authors concluded that the anchor nail displacement was related to the device technique and planned to reopen the clinical trial.

Unlike other solutions, the CardioMech system (Figure 8C) protocol implanted only a piece of chordal to treat MR, with metal pieces connected at both ends of the suture to anchor the leaflet and papillary muscle. Rinaldi reported three clinical trials of the CardioMech system at the TCT 2021 conference, and all three patients with P2 region prolapse had a reduction in MR from 4+ to <2+ after successful implantation of one chord, with no serious adverse events occurring during the operation. However, postoperative dislodgement of the leaflet anchor nail occurred in all three patients and was ultimately unsuccessful.

The Neochord NeXuS system was a product based on the DS1000 for transfemoral/interatrial septal access that accessed the left atrium through a large 28F sheath via the femoral vein, captured the leaflet under the guidance of TEE and confirmed by a fiber optic sensor, and finally achieved multiple chords attached to a papillary muscle metal screw anchor. Currently, Latib et al. (2022) completed the FIH clinical trial with the Neochord NeXuS system in 2021 and reported 6-month follow-up results. Two pairs of artificial chords implanted in the leaflet and anchored to the anterior papillary muscle in a 55-year-old patient with prolapsed MR in the P2 region successfully reduced MR and maintained mild or trace MR at 1/3/6 months with firm anchoring of the papillary muscles without displacement.

Transcatheter chordae repair devices might be a viable and feasible option for the treatment of isolated single-leaflet prolapse in the future, with a good risk-benefit ratio. However, the technique and procedures were more complex with many steps and were still in the early stages of development, requiring stable devices, long-term follow-up, and more cases to confirm these preliminary results.

## 5 Artificial chordae rupture cases and failure factors

Transapical implantation of ePTFE chordae under beating heart condition was a minimally invasive interventional therapy with a very low rupture rate and proven durability and long-term results in a 25-year study published in 2013 (David et al., 2013), showing a bright prospect for the treatment of DMR. However, in early clinical trials, there were still reports of artificial chordae rupture cases, whether in traditional surgery or intervention, (Mori et al., 2017). Although rare, the rupture of the artificial chordae might cause recurrent mitral regurgitation, endangering patients’ health. We reviewed the cases of recurrent MR caused by rupture of ePTFE artificial chordae and discussed the possible causes of rupture.

**FIGURE 9:**
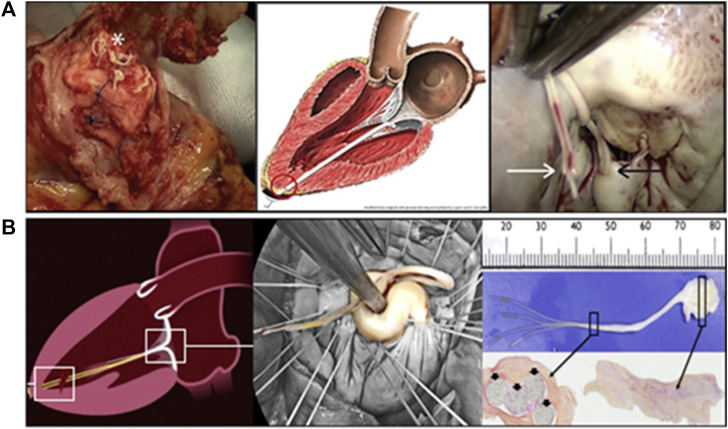
**(A)** A rupture in an implanted NeoChord below the knot and the pledget (Heuts et al., 2019). **(B)** Surgical view and histologic analysis of the resected ruptured neochords (Grinberg et al., 2019a).

Heuts et al. (2019) reported in 2019 that a female patient’s implanted artificial chordae ruptured at the top of the apex knot (Figure 9A) during cycling 5 months after NeoChord treatment, resulting in acute MR. Grinberg et al. (2019a) reported two cases of chordae ruptured 1 year and 3 year after the implantation of the NeoChord DS1000 system in 2019. Both of them underwent second surgeries (Figure 9B). Bortolotti presented one patient with 2 pairs of CV-5 ePTFE implanted in the anterior leaflet that ruptured after 11 years postoperatively. In one case, only minimal calcification was observed, and the chordal rupture was therefore considered to be most likely related to weakening of the ePTFE by collagen infiltration and fatigue-induced lesions (Bortolotti et al., 2012a).

The long-term durability of transapical artificial chordae implantation was still unknown, so it was very important to understand the potential mechanism of chordal rupture.

### 5.1 Analysis of the force difference of artificial chordae between different anchorage positions

The durability and long-term effects of artificial chordae were closely related to the amount of force applied to them, and excessive force could lead to chordae rupture. The anchoring position and the length of the artificial chordae influenced their force. Surgical thoracotomy and transcatheter artificial chordae tended to be anchored to the leaflet and papillary muscles, whereas transapical chordae implantations tended to be leaflet and the apex. Since artificial chordae implantation led to post-reconstruction shrinkage of the left ventricle, the length of the artificial chordae was often shortened somewhat from the optimal length to avoid recurrent MR (Bortolotti et al., 2012b). For apical anchoring, it was often clinically tightened by 5% from the optimal length; for papillary muscle-anchored chordae, it was shortened by 1–2 mm (Kasegawa et al., 1994).

Caballero et al. (2020) analyzed the effect of transapical anterolateral and posterolateral approaches and length on the force of artificial chordae through computer simulation. Their results showed that the forces on the artificial chordae with the anchoring position in the anterolateral apical region were generally greater than those in the posterolateral region, reaching more than 80% at the peak systolic period. Therefore, the postero-lateral approach was preferred for transapical minimally invasive instrumentation. Four artificial chordae were implanted in a model of severe MR due to prolapse the P2 region, and the total force was 2.68 N when the chordae were at their optimal length, which increased significantly with 5% tension, reaching a maximum total force of 12.2 N and a maximum force of 4.2 N for a single chord. Sturla et al. (2015) investigated the effect of the length of the artificial chords anchored to the papillary muscles on their force. The optimal length and the maximum force of 2 mm tightening were 1.31 and 1.62 N, respectively; when two artificial chordae were implanted, the maximum total force was 1.54 N for the optimal length and 1.92 N for the 2 mm tightening. According to the simulation force analysis, the force of the artificial chordae anchored at the apex was significantly higher than that of the papillary muscle, which might pose a challenge for the long-term durability of the artificial chordae implanted by transapical devices. Grinberg et al. (2019b) measured forces on the neochordae when implanted apically using the DS1000 system in humans. After all the neochordae were implantated, tracked the neochordae respectively. First, the neochordae implanted in the ideal location (in the middle of the prolapse) were tracked alone until the best TEE control was obtained. The tension was measured at between 0.7 and 0.9 N, and the oscillation in the amplitude of tension of about 13% that followed the respiratory cycles; second, the other neochordae were then tracked one by one with a single screw to achieve an equivalent tension on all neochordae, the tension of the neochordae tracked first was reduced from 0.8 N to 0.2–0.3 N when 4 neochordae were put in tension. The tension (F = 0.98 ± 0.08 N) of all neochordae was higher than the previous tension (F = 0.8 N) of the tracked neochordae; then all neochordae were tracked together under TEE control until the optimal condition was reached. When the optimal TEE results were obtained, the tension of all neochordae was reduced to 0.87 ± 0.07 N, about 12% ± 2% decrease; and finally all the neochordae were fixed at the apex of the left ventricle at the optimal length. During all the steps, neochordae tension, electrocardiogram, radial blood pressure, 3-D TEE, and surgical view were recorded. The author said in the section of study limitation that relative to the resistance, the length and direction of tension of the neochordae (from the leaflet free edge to the apex) were more important, which were different from those of the native chordae.

### 5.2 Differences in chordae implantation techniques in the leaflet

**FIGURE 10:**
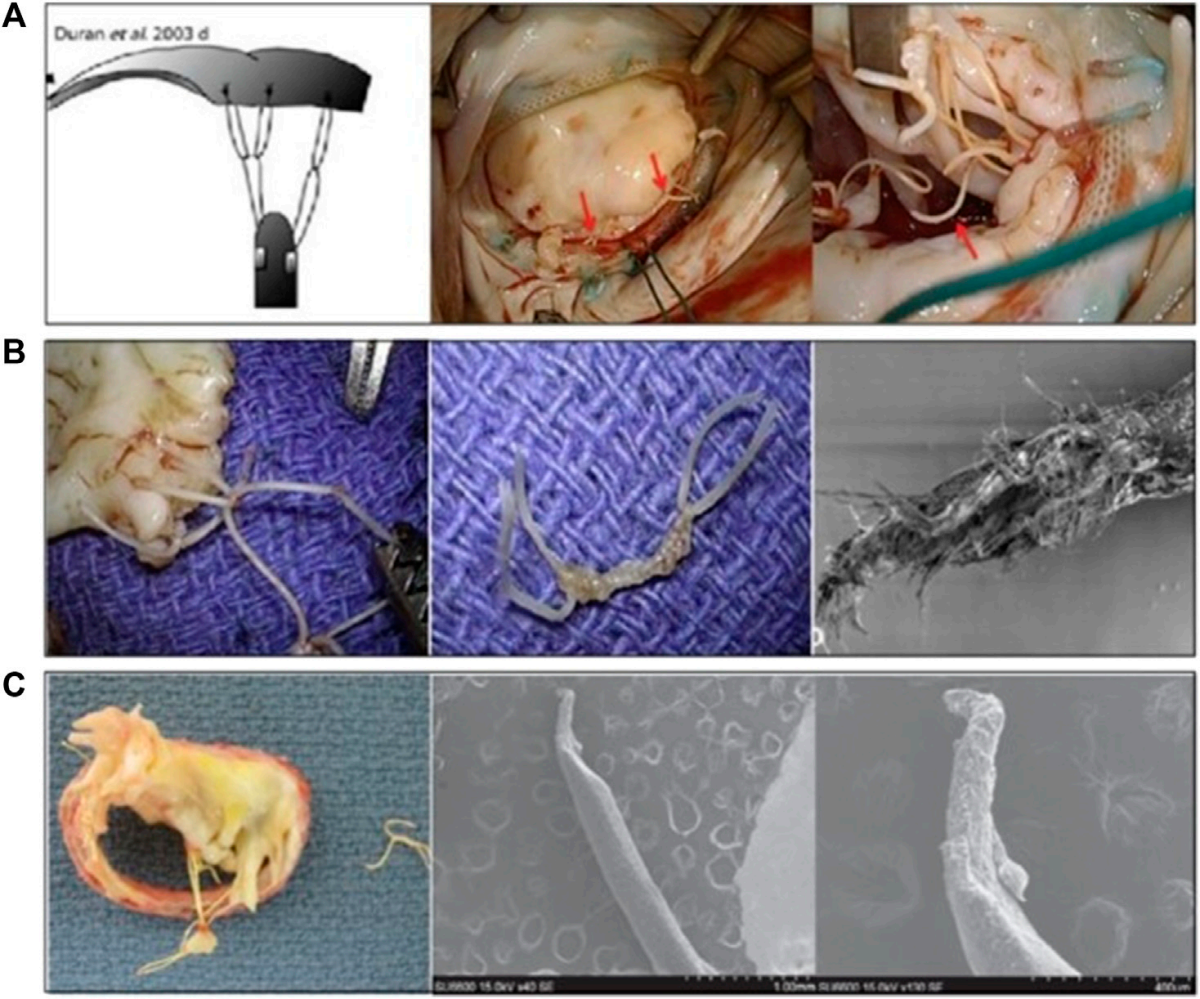
**(A)** The “loop” artificial chordal Implantation Technology (Duran and Pekar, 2003; Kudo et al., 2014), **(B,C)** Macro and SEM images of the partial fracture of the ePTFE loop (Castillo et al., 2013; Kudo et al., 2014).

Differences in chordae implantation techniques might have a significant impact on the durability of the ePTFE artificial chordae. We found several cases of chordae rupture in the “loop” implantation technique (Figure 10A) (Duran and Pekar, 2003; Kudo et al., 2014), probably due to frictional damage to the suture caused by the relative motion of the two loops during the cardiac cycle. This frictional rupture often occured in the early and middle stages. Castillo described a case in which the “loop” technology was used for repair. Ultimately, the CV-5 ePTFE suture ruptured, which was analyzed by scanning electron microscopy (SEM) and found to be weakened at the point of contact between the two suture rings, which the author believed was caused by relative friction (Figure 10B) (Castillo et al., 2013). Kudo reported a case of CV-5 ePTFE suture loop rupture, which was found to be thinning by SEM, with a rupture time of 3 years, also using the “loop” technique (Figure 10C) (Kudo et al., 2014). Yamashita and Skarsgard (2011) reported two cases of early CV-5 ePTFE rupture at 14 and 2 months postoperatively, respectively, using the “loop” technique in both patients.

### 5.3 Calcification

**FIGURE 11:**
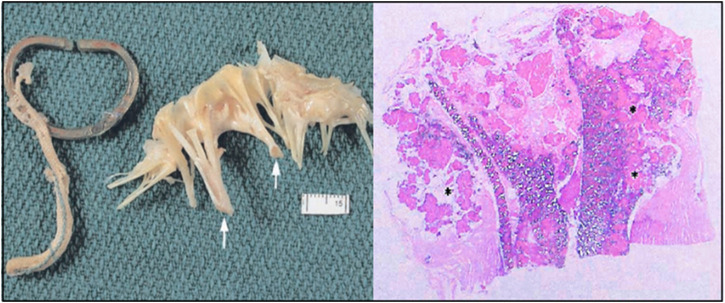
The anterior leaflets showed thickening chordae tendineae (white arrows). Longitudinal sections through the ruptured ePTFE suture. Large areas of mineralization were seen within the graft interstices and in the areas surrounding the graft (asterisk) (stain: hematoxylin and eosin, tissue viewed with polarized light, original magnification = ×1.6) (Butany et al., 2004).

Calcification might be one of the causes of the late rupture of ePTFE artificial chordae. Butany et al. (2004) reported a case of rupture after 14 years postoperatively. Histopathology showed that the ePTFE sutures ruptured due to calcification, but no inflammatory cells were found inside and around the sutures (Figure 11). Luthra et al. (2022) reported a 76-year-old woman who presented with severe mitral regurgitation 6 years after Neochord artificial chordae repair with Gore-Tex, the explanted sutures were found to be ruptured and stiff. Electron microscopy revealed microstructural disruption with extensive calcium infiltration at the site of rupture. Farivar et al. (2009) reported a case of rupture of two pairs of CV-5 chords after 11 years after the implantation in which the ePTFE was thickened and hardened. Coutinho et al. (2007) reported two cases of rupture; one patient had two pairs of CV-5 sutures implanted with a rupture time of 6 years and an unknown cause of rupture; the other patient also had two pairs of CV-5 sutures implanted with 1 pair of chords ruptured after 11 years due to calcification.

### 5.4 Specification of sutures

The rupture of the ePTFE artificial chordae might also be related to the suture specification. First, in all of the above cases, the sutures that ruptured were mainly CV-5 ePTFE sutures. Mutsuga reviewed the failure cases of using ePTFE artificial chordae from 2002 to 2020. One (0.5%) of 186 patients using CV-4 ruptured at 78 months. In addition, 6 (3.9%) of 154 patients using CV-5 ruptured at 44–201 months. The durability of CV-4 specification ePTFE suture might be better than CV-5 (Table 4) (Mutsuga et al., 2022). Therefore, there was a growing preference among physicians to use CV-4 with a thicker line diameter and stronger mechanical properties, and to increase the number of sutures to distribute the force on a single suture (David et al., 2020).

**TABLE 4:** Distribution of use of expanded polytetrafluoroethylene suture on each mitral valve (Mutsuga et al., 2022).

Suture	AML	PML	AML + PML	Others
CV-4				
Ruptured ePTFE	1	0	0	0
Number	186	238	116	84
patients	45	59	18	17
Rupture rate, %	0.5	0	0	0
CV-5				
Ruptured ePTFE	6	9	0	0
Number	154	394	96	176
patients	41	95	16	23
Rupture rate, %	3.9	2.3	0	0
*p*-value	.03	.019	…	…

AML, anterior mitral leaflet; PML, posterior mitral leaflet.

### 5.5 Other factors

There were also some cases where fracture factors were not described. Nakaoka reported a patient with three pairs of CV-5 ePTFE implanted in the anterior leaflet, in which one pair of ePTFE ruptured after 7 years, and the suture was thickened (Nakaoka et al., 2017). Yeo described a patient with chordal rupture at 4 months postoperatively (Yeo et al., 1998), and Lam reported a case with an unknown rupture time, neither of which mentioned the cause of the rupture (Lam et al., 2004).

## 6 Conclusion and outlook

At present, the use of ePTFE artificial chordae to resolve mitral valve prolapse caused by extended or ruptured native chordae has become a mature method with a clinical history of more than 35 years, providing stable valve function and clinical outcomes in most patients with DMR. The long-term durability of ePTFE played a key role, which was closely related to its physicochemical properties and biocompatibility. Although this technology was safe and effective, there were still cases of ePTFE artificial suture rupture occurring due to chordae calcification, friction, surgical injury, and excessive force. Therefore, when performed artificial chordae repair, some measures could be taken to reduce the probability of this adverse event, such as 1) paying attention to chordae implantation and suturing techniques to avoid frictional damage to the sutures; 2) increasing the number of chords to reduce the force distributed on each suture; and 3) using CV-4 instead of CV-5, which had a thicker string diameter and stronger mechanical properties.

Artificial chordae interventional devices had gradually become a substitute therapy for high-risk DMR patients, providing physicians with a variety of options. The two different types of devices had their own advantages and disadvantages. Among them, the transapical chordae repair devices had more clinical data, relatively simple technique and operation, and a high success rate, but it was traumatic, and the force on the chordae was greater than the papillary muscle anchoring. Transcatheter chordae repair devices were in the early investigational phase with limited clinical data. Their advantages include less trauma and anchoring of the chordae to the papillary muscle, which was closer to the physiologic and anatomic structure. However, they had narrow indications and anatomical limitations, and the technique and operation were relatively complex. In the future, clinical studies with larger sample size and longer follow-up are necessary to confirm the long-term outcomes and patient selection criteria.

As an important part of the interventional mitral valve treatment technology, transcatheter chordae repair technology will continue to be a mainstream development direction in the field of interventional mitral valve treatment in the future, which has unparalleled advantages over interventional mitral valve replacement such as:

(1) More physiological.
(2) Less impact on mitral valve hemodynamics.
(3) No interference with future secondary surgical procedures.
(4) Feasibility for low-risk, low-grade patients.

The continued emergence of interventional transcatheter chordae repair devices via the femoral vein also meant that a solid step had been taken in this technical field and that it still had a wild prospective.

For the patients with prolapse but no significant dilatation of the valve ring, whose cardiac function was still good, interventional transcatheter chordae repair devices were the most beneficial for them at that time.

However, in high-risk patients with severe mitral regurgitation leading to the stage of heart failure, the lack of clinical experience with simple interventional artificial chordae repair and the existence of distant recurrent regurgitation risk, which to some extent limits the application of artificial chordae, but we still have reason to believe that with the accumulation of experience and the progress of technology, the complete intervention of mitral valve repair will no longer be an unattainable dream.
